# Degrading permafrost river catchments and their impact on Arctic Ocean nearshore processes

**DOI:** 10.1007/s13280-021-01666-z

**Published:** 2021-11-30

**Authors:** Paul J. Mann, Jens Strauss, Juri Palmtag, Kelsey Dowdy, Olga Ogneva, Matthias Fuchs, Michael Bedington, Ricardo Torres, Luca Polimene, Paul Overduin, Gesine Mollenhauer, Guido Grosse, Volker Rachold, William V. Sobczak, Robert G. M. Spencer, Bennet Juhls

**Affiliations:** 1 Dept of Geography & Environmental Sciences, Northumbria University, Newcastle upon Tyne, NE1 8ST UK; 2 Alfred Wegener Institute Helmholtz Centre for Polar and Marine Research, Telegrafenberg A45, 14473 Potsdam, Germany; 3 University of California, Santa Barbara, UCEN Rd, Goleta, CA 93117 USA; 4 Alfred Wegener Institute Helmholtz Centre for Polar and Marine Research, Am Handelshafen 12, 27570 Bremerhaven, Germany; 5 Plymouth Marine Laboratory, Prospect Place, Plymouth, PL1 3DH UK; 6 Institute of Geosciences, University of Potsdam, Potsdam, Germany; 7 Department of Biology, College of the Holy Cross, 1 College St, Worcester, MA 01610 USA; 8 Florida State University, 303 Oceanography Building, Tallahassee, FL 32306 USA

**Keywords:** Arctic rivers, Carbon cycle, Carbon fluxes, Erosion

## Abstract

Arctic warming is causing ancient perennially frozen ground (permafrost) to thaw, resulting in ground collapse, and reshaping of landscapes. This threatens Arctic peoples' infrastructure, cultural sites, and land-based natural resources. Terrestrial permafrost thaw and ongoing intensification of hydrological cycles also enhance the amount and alter the type of organic carbon (OC) delivered from land to Arctic nearshore environments. These changes may affect coastal processes, food web dynamics and marine resources on which many traditional ways of life rely. Here, we examine how future projected increases in runoff and permafrost thaw from two permafrost-dominated Siberian watersheds—the Kolyma and Lena, may alter carbon turnover rates and OC distributions through river networks. We demonstrate that the unique composition of terrestrial permafrost-derived OC can cause significant increases to aquatic carbon degradation rates (20 to 60% faster rates with 1% permafrost OC). We compile results on aquatic OC degradation and examine how strengthening Arctic hydrological cycles may increase the connectivity between terrestrial landscapes and receiving nearshore ecosystems, with potential ramifications for coastal carbon budgets and ecosystem structure. To address the future challenges Arctic coastal communities will face, we argue that it will become essential to consider how nearshore ecosystems will respond to changing coastal inputs and identify how these may affect the resiliency and availability of essential food resources.

**Supplementary Information:**

The online version contains supplementary material available at 10.1007/s13280-021-01666-z.

## Introduction

**Fig. 1:**
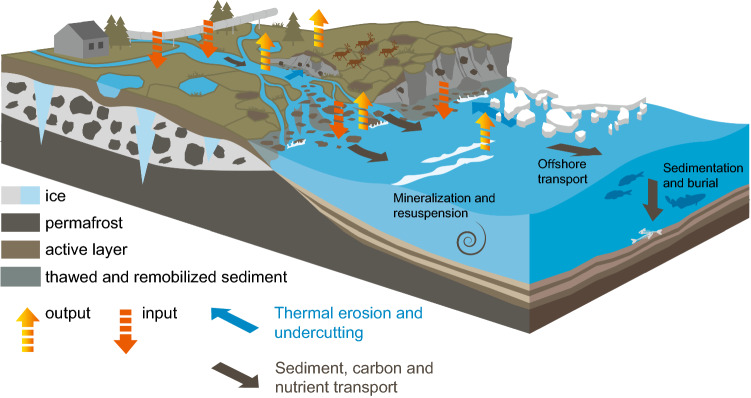
Future response of nearshore environments to climate change, and potential impacts to ecosystem processes and coastal biogeochemistry. Terrestrial permafrost thaw causes landscape collapse and changing resources, affecting terrestrial infrastructure (drawn as house and pipeline) and distributions of food and traditional lands (represented by reindeer on land). Permafrost thaw on land can affect terrestrial gas fluxes, or be mobilised into freshwaters, affecting OC reactivity and carbon budgets from the river, delta or gulf regions (input/output arrows). Changing terrestrial OC supply (black arrows) may influence nearshore carbon, nutrient budgets, and food web dynamics, altering air-sea gas fluxes (coastal inputs/outputs/processes) or essential coastal food resources (represented as fish/whale). Drawn by Yves Nowak (AWI)

The Arctic region is experiencing unprecedented change to its physical environment in response to global climate disruptions, causing a multitude of social, geopolitical and ecosystem instabilities. One of the greatest challenges facing the region is due to the loss of permafrost-perennially frozen ground that remains at or below 0 °C for at least two consecutive years (Van Everdingen 2005). Almost five million people live and rely on permafrost ground across the Arctic (4.9 million in 2017; Ramage et al. 2021) and are susceptible to on-going surface permafrost thaw in response to warming Arctic air temperatures (Biskaborn et al. 2019). Loss of terrestrial permafrost causes direct damage to essential infrastructure and impacts upon the livelihoods and culture of local people (Ford and Pearce 2010; Fig. 1). Food and water security have been, and will be, negatively impacted by changes in lake, river and shore-fast ice, as well as permafrost in many Arctic regions (Strauss et al. 2021a). These changes have disrupted access to herding, hunting, and fishing grounds (Fig. 1), and caused the instability of agricultural land (IPCC 2019).

**Fig. 2:**
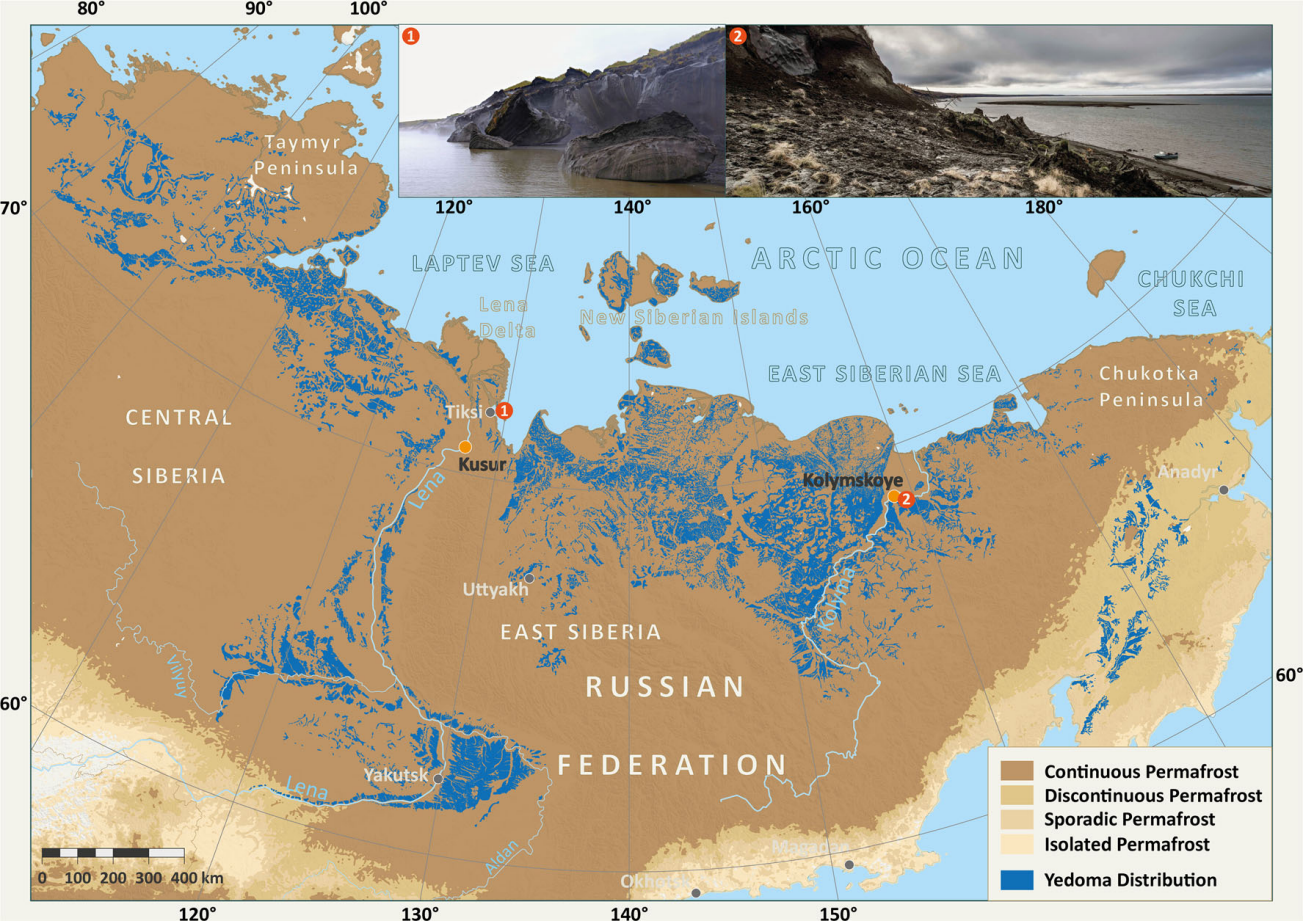
Permafrost (after Obu et al. 2019) and Yedoma permafrost (Strauss et al. 2021a, b) distribution (map) with two sites of rapidly eroding cliffs as examples. Site 1: Mamontovy Khayata cliff on the Bykovsky Peninsula near the coast of the Lena Delta (credit: P.P. Overduin) and, Site 2: the Duvanny Yar exposure (site 2) on the Kolyma river (credit: A. Stubbins). Freshwater discharge measurement stations at Kusur (Lena) and Kolymskoye (Kolyma) are shown (orange dots). Drawn by S. Laboor

Terrestrial permafrost thaw across river catchments can liberate peat and permafrost-derived OC from soils to inland aquatic ecosystems (Frey and Smith 2005; Wild et al. 2019), modifying stream food web dynamics by changing nutrient or carbon availabilities to aquatic microorganisms (Slavik et al. 2004). Permafrost, specifically ice- and organic-rich Yedoma permafrost (Fig. 2 insets, Yedoma definition in Strauss et al. 2021a, b), has been shown to be of ‘high quality’ for microbial communities (Strauss et al. 2015, 2017, 2021a, b; Jongejans et al. 2018; Haugk et al. in review) likely due to its rapid formation limiting prior processing during the Late Pleistocene. Once mobilised into inland waters, permafrost-derived OC can be rapidly utilized by aquatic microorganisms, increasing bulk OC degradation rates in riverine and coastal Arctic water incubations (Vonk et al. 2013; Drake et al. 2015; Mann et al. 2015) and potentially enhancing riverine CO_2_ losses from river basins (Vonk and Gustafsson 2013; Drake et al. 2018a, b; Fig. 1).

Permafrost OC inputs to Arctic headwaters have been shown to be preferentially utilised by aquatic microorganisms, leading to patterns of decreased permafrost contributions in OC pools with increased water residence times (Mann et al. 2015). In addition, a general pattern of decreasing dissolved OC (DOC) reactivity has been demonstrated with increasing retention time of waters across diverse global river catchments, highlighting a universal decline in DOC reactivity along the aquatic-ocean continuum (Catalán et al. 2016; Soares et al. 2019). Any hydrologic changes, such as increases to river discharge or extreme flow events, causing shorter transit times would therefore result in OC bypassing headwater streams and being metabolized in mainstream and nearshore coastal waters, in agreement with the pulse-shunt concept (Raymond et al. 2016).

Arctic hydrological cycles are already intensifying. Pan-Arctic freshwater runoff rates to the Arctic Ocean have increased from 3900 ± 390 km^3^ in 1980–2000 to 4200 ± 420 km^3^ by 2000–2010 (Haine et al. 2015). Global climate model projections indicate that future freshwater runoff will continue to increase and that the rate of increase may accelerate over much of the Arctic during the coming decades (Haine et al. 2015; Brown et al. 2019). Combined hydrologic models informed using climate projections estimate freshwater discharge increases of ~ 25 to 50% to the Laptev and East Siberian Shelf by 2100 (Arnell 2005; Shiklomanov et al. 2013; van Vliet et al. 2013; Koirala et al. 2014; Andreson et al. 2020; Wang et al. 2021). Higher rates of continental freshwater runoff patterns will alter the distribution of terrestrial OC within river networks, and likely deliver greater quantities of OC from degrading river catchments to the coastal ocean. This has the potential to alter the availability of nutrients and carbon across the nearshore and modify the physiochemical environment (e.g., light penetration or carbonate system).

Here, we examine how future projected increases in runoff and permafrost thaw from two permafrost-dominated Siberian watersheds—the Kolyma and Lena, may alter carbon turnover rates and OC distributions through river networks. We present experimental results from the Kolyma River examining how rates of OC degradation in riverine carbon pools will shift with compositional changes associated with permafrost thaw OC. We then explore potential for future permafrost thaw and hydrological intensification in these basins to alter terrestrial OC loads to East Siberian Arctic Shelf (ESAS) nearshore waters, by scaling our findings to the Lena River. We finally explore potential for future permafrost thaw and hydrological intensification in these basins to alter terrestrial OC loads to East Siberian Shelf nearshore waters. We conclude that there is a substantial paucity of information on how the rapidly changing terrestrial environment may affect coastal ecosystems and processes, and that future research and modelling work is needed to predict how ecosystem functioning and essential food webs may change under future scenarios.

## Materials and methods

### Study region

Our study focused on the Lena and Kolyma River catchments, two great watersheds that together comprise 19% of the pan-Arctic watershed and drain a watershed area of 3.11 million km^2^ from the permafrost-dominated continental region into the ESAS. The shallow ESAS (average depth 58 m; Jakobsson 2002) represents a quarter of the Arctic shelf area (Shakhova et al. 2010) and is particularly vulnerable to changing inputs of terrestrial OC, with extreme regional climate warming already causing these Siberian terrestrial permafrost-rich watersheds to thaw (Graversen et al. 2008; Shakhova et al. 2010).

The Lena and Kolyma rivers account for a combined annual terrestrial OC flux of 7.0 to 9.4 TgC year^−1^ (Holmes et al. 2012; McClelland et al. 2016; Juhls et al. 2020), which is approximately 17 to 28% of total terrestrial OC loads to the Arctic Ocean (Raymond et al. 2007). Large quantities of permafrost OC are stored in Pleistocene Yedoma deposits (Strauss et al. 2017), which when degraded or eroded, can represent hotspots of old terrestrial OC release to river catchments (Wetterich et al. 2020). Both the Kolyma and Lena River watersheds contain relatively similar coverage in Yedoma deposits, representing 7.7% of the watershed area in the Kolyma watershed area, and 3.5% of the Lena. Examples of such rapidly eroding Yedoma riverbanks include the Sobo-Sise cliff on the Lena River (Fuchs et al. 2020) and the Duvanny Yar cliff (Fig. 2 inset) on the Kolyma River (e.g., Strauss et al. 2012). Riverine OC loads to coastal waters from both rivers are predominantly (> 80%) in the dissolved form. The composition of the dissolved OC pools in the Kolyma and Lena Rivers are similar with comparable fractions of hydrophobic acids, transphilic acids, and hydrophilic organic matter as a percentage of total OC concentrations (Table 1; Mann et al. 2016). Additionally, the overall aromaticity of the OC pools are comparable, as inferred from organic matter absorbance measurements (specific ultraviolet absorbance; Mann et al. 2016). The two river catchments differ significantly in the type and morphometry of their estuaries, with the Lena River feeding into an extensive delta before reaching the coastal ocean. The Kolyma, by contrast, runs directly through a gulf feeding directly onto the East Siberian Sea shelf (Fig. 2). Coastal erosion also delivers large amounts of OC into the nearshore, for example from the Mamontovy Khayata coastal cliff on the Bykovsky Peninsula (Fig. 2) (Lantuit et al. 2011; Rolph et al. 2021) or other Yedoma coastal segments along the Laptev Sea coast (Günther et al. 2013; Strauss et al 2021b).

**Table 1:** First-order OC degradation rates (day^−1^) and OC lifetimes for each fraction determined in our experiments (Rapid OC) and in previous literature (Slow OC)

	OC degradation rate (day^−1^)	OC lifetime (year^−1^)
Rapid OC fraction (*n* = 34)		
Mean	0.0139	0.20
Median	0.0095	0.29
Stdev	0.0152	0.18
Min	0.0022	1.25
Max	0.0632	0.04
Slow OC fraction (*n* = 18)		
Mean	0.0029	0.95
Median	0.0024	1.14
Stdev	0.0021	1.34
Min	0.0013	2.11
Max	0.0098	0.04

### Contemporary river OC degradation rates

We measured river OC degradation rates (*n* = 34) using oxygen loss measurements on Kolyma lower mainstem waters (within 100 km of river mouth), collected during the summers of 2011 and 2012 (Table S1). Water samples were also collected from under-ice (May) and during the spring freshet (June) during 2012 from the Kolyma mainstem. Unamended biological oxygen demand (BOD) assays (i.e., waters were not seeded or primed) were run over a 5-day period on unfiltered waters at room temperature (~ 20 °C; Jiao et al. 2021). Waters were slowly decanted into triplicate 300 mL glass BOD bottles and total oxygen concentrations measured using self-stirring optical optode oxygen probes (YSI, ProOBOD, ± 0.1 mg L^−1^) after 0, 1 and 5 days. BOD assays measure the amount of dissolved oxygen used by microbial communities during degradation of OC and are converted to OC carbon concentrations using a commonly applied respiratory quotient of 1 (assuming a ratio of 1 between CO_2_ production and O_2_ consumption). BOD assays are sensitive to small changes in the OC pool and are suitable for capturing OC rates associated with rapidly available and fast turnover OC pools. As such, rates determined using this method are henceforth considered to represent a *rapid OC pool*.

To supplement our OC degradation measurements, we collated our results with previously published rates determined in Kolyma River mainstem waters (Mann et al. 2012, 2015; *n* = 18, Table S1). Samples from these studies were collected in the Kolyma River across a similar region of the lower river catchment (approximately 100 km of the mouth: site locations Table S1), during the freshet and late autumn periods. These studies calculated OC degradation rates using direct dissolved OC (DOC) losses measured over a 28-day incubation period to provide insights into a slower OC fraction turn over approximately monthly timescales. Rates determined using this method are henceforth considered to represent a *slow OC pool*.

Direct and inferred OC loss measurements from all studies were fitted to an exponential decay to determine OC degradation rates (*k*) from incubation experiments:

1 $${\text{OC}}_{t} = {\text{ OC}}_{{{\text{init}}}} e^{ - kt}$$

where OC_*t*_ represents the OC concentration at time (*t* in days), OC_init_ represents the initial OC concentration and *k* the degradation rate (day^−1^).

OC degradation rates (*k*) were corrected to the in-situ water temperature measured at the study site during sampling (or other as stated below), using a form of the Arrhenius equation:

2 $$k_{T} = \frac{{k_{20} }}{{q_{10}^{{\frac{{\left( {20 - {\text{Temp}}} \right)}}{10}}} }}$$

where *k*_T_ is the corrected OC degradation rate (day^−1^), *k*_20_ the degradation rate in incubations at 20 °C (from Eq. 1) and Temp the measured in-situ water temperature (°C) at the time of sampling. *q*_10_ is the temperature coefficient which was assumed to be 2.0 (following estimates from Wickland et al. 2012; Catalán et al. 2016). To allow direct comparisons with other studies which present terrestrial OC lifetimes in reciprocal time units (the time by which an OC pool [*X*] is degraded to a value equal to [*X*]/*k*_T_) as per Hansell (2013) we additionally present these alongside measured rates (day^−1^).

### Freshwater discharge measurements

River discharges associated with degradation experiments (Table S1) were determined using data from the Arctic Great Rivers Observatory website (Shiklomanov et al. 2021). Discharge measurements from gauging stations at Kolymskoe, located approximately 160 km upstream of our sampling sites were used (Fig. 2). Adjustments were made to account for the transit time of water between the gauging station and our lower Kolyma River sites by assuming river velocities of 1.5 m s^−1^ as in Holmes et al*.* (2012).

To assess past trends and contemporary discharge rates for the Kolyma and Lena rivers, we analysed discharge measurements from gauging stations at Kolymskoe (1978–2020) and Srednekolymsk (1927–2016, with gaps) from the Kolyma River basin, and at Kyusur (1936—2020) on the Lena river (Fig. 2). Both were monitored by the Russian Federal Service for Hydrometeorology and Environmental Monitoring (Roshydromet). Climate projections estimate mean annual runoff increases of ~ 50% (± 25%) in the Kolyma River and 25% (+ 25%/− 20%) for the Lena River by the end of the twenty-first century (Arnell 2005; Shiklomanov et al. 2013; van Vliet et al. 2013; Koirala et al. 2014). To estimate future discharge rates, we applied these projected increases relative to a baseline period of 1971–2000 from both rivers.

### Impact of permafrost thaw OC on freshwater degradation rates

We conducted an experiment to assess if inputs of permafrost thaw OC, and the associated change in aquatic carbon composition, cause changes to bulk OC degradation rates. We specifically examined if the compositional changes alone, independent from concentration changes, cause changes to carbon turnover.

Frozen ice-wedge samples were collected from the Duvanny Yar exposure within the Kolyma River Basin during early September 2013 (Fig. 2). Yedoma deposits at Duvanny Yar accumulated between ∼ 40 and 13 ky BP (Vasil’chuk et al. 2001) and are believed to be of polygenetic origin (Strauss et al. 2012). Total average ice content is approximately 75% by volume (35 wt% for ground ice, plus about 50 vol% for ice wedges) and total OC content averages 1.5 ± 1.4 wt% (Strauss et al. 2012). Ice wedge thaw waters carry old terrestrial OC from Yedoma exposures (19,350 to 29,400 years; Vonk et al. 2013; Spencer et al., 2015) directly into the Kolyma River mainstem.

Combined ice-wedge and permafrost samples were chiselled from the cliff and kept cool and dark until laboratory preparation (< 48 h). A bulk Kolyma River water sample was collected upstream of the exposure, representing mainstem waters unaffected by Duvanny Yar permafrost thaw subsidies in our experiment. In the laboratory, ice-wedge and permafrost were thawed in a double acid-rinsed glass container, before filtration through glass fibre filters (pre combusted Whatman GF/F, nominal pore size of 0.7 µm). Filtration removes a proportion of the microbial community, but this approach has been shown to provide comparable results to degradation experiments using a starting inoculum (Vonk et al. 2015). Kolyma mainstem waters were filtered in an identical manner. DOC concentrations were then measured (as below) in the Kolyma River (4.8 ± 0.5 mg/L; *n* = 6) and ice-wedge mix waters (86.4 ± 2.1 mg/L; *n* = 6), and the ice-wedge waters diluted with Milli-Q waters to match the DOC concentration of the Kolyma River waters.

A series of sample mixtures were then produced containing 0, 1, 10, 25, 50, 75, 99% final contributions of ice-wedge Kolyma River waters (Average initial concentrations = 5.8 ± 0.7 mg/L; *n* = 27). A minimum of two incubations were run per mixture. Samples were stored dark at room temperature (approximately 20 °C) and agitated daily to ensure sample mixing. Duplicate vials were sacrificed after 14 and 28-days, filtered as above and then acidified with H_3_PO_4_ until pH 1–2 and kept in the dark at 4 °C until analysis. DOC concentrations were measured using the combustion catalytic oxidation method (Shimadzu TOC-L, ± 0.1 mg/L). The differences in DOC concentrations over 28-days were calculated and assigned to turnover rates of the *slow* OC pool as above. The differences in DOC concentrations over 14-days were used to determine a separate *fast* OC pool.

To supplement our permafrost experimental results, we collated published OC degradation measurements from Arctic River waters amended with Yedoma additions to examine the impact of permafrost thaw on inland waters (*n* = 39; Table S2).

## Results

### Terrestrial OC degradation rates in Arctic freshwaters

Natural mean degradation rates in the rapid OC fraction measured using short-term oxygen loss measurements were 0.0139 day^−1^ (s.d. ± 0.0152 day^−1^), corresponding to lifetime estimates of 0.20 year^−1^ (± 0.18 year^−1^; Table 1) for this fraction. Mean degradation rates in the slow turnover OC pool were lower (0.0029 ± 0.0021 day^−1^), with correspondingly longer lifetime estimates of 0.95 year^−1^ (± 1.34 year^−1^; Table 1) for this fraction. Our mean (0.0029 day^−1^) and median (0.0024 day^−1^) bioactivity rates in the slow OC pool compare closely yet slightly lower than the median *k* value of 0.0034 ± 0.0219 day^−1^ reported from 46 separate global river systems (Catalán et al. 2016).

### River hydrology patterns

**Fig. 3:**
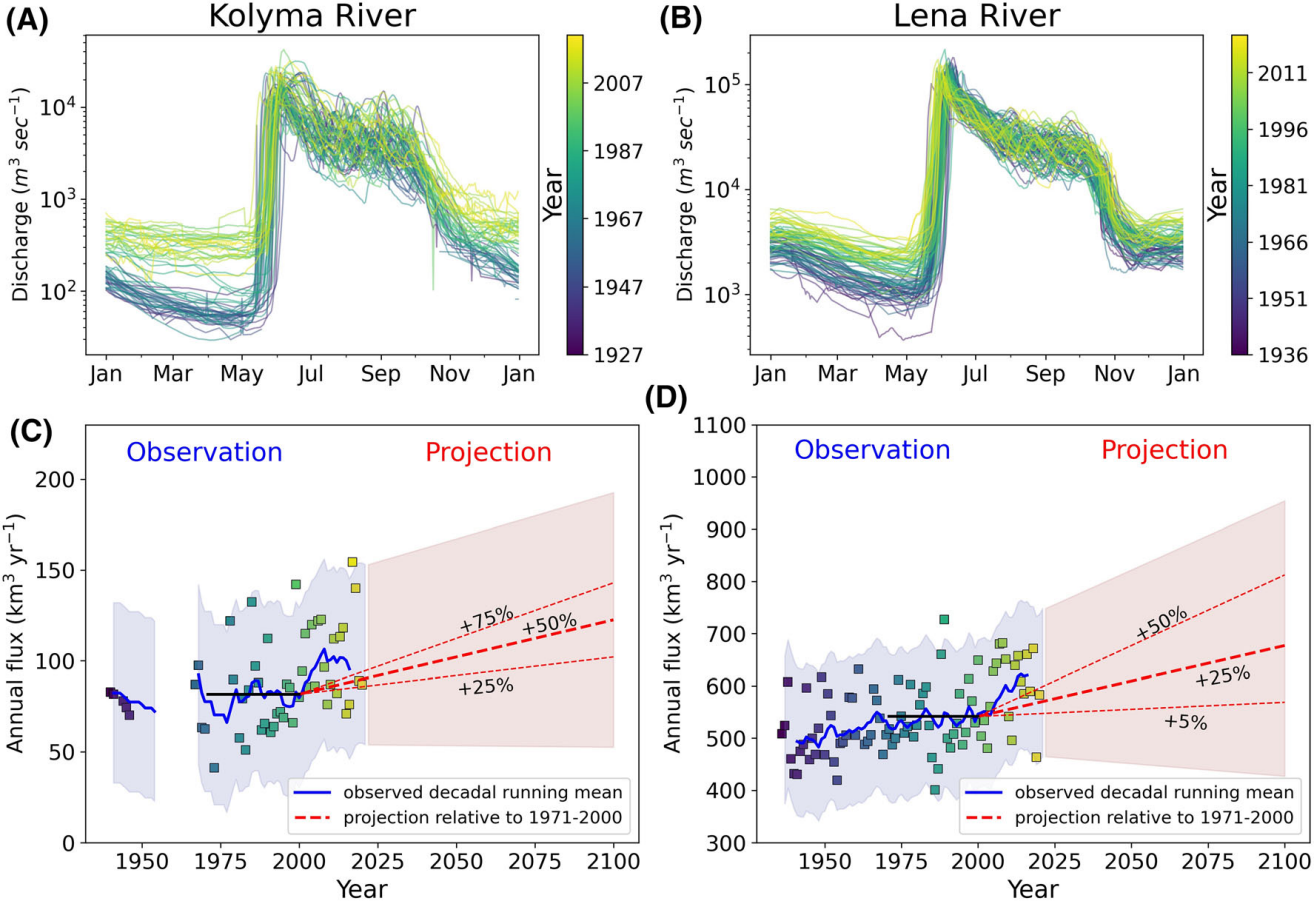
Upper panel: Hydrograph of **a** Kolyma River for all years from 1927 to 2020 and **b** Lena River from 1936 to 2020. Lower panel: Observed and projected freshwater discharge (km^3^ year^−1^) for **c** the Kolyma and, **d** Lena Rivers. Blue line on each plot represents the decadal running mean and filled blue colour the second standard deviation of the observed discharge. Red dashed lines show different projection scenarios to 2100 against the baseline period from 1971 to 2000 (black line). Filled red colour indicates the observed second standard deviation applied on chosen minimum and maximum projection scenarios

The overall load and timing of freshwater discharge from the Kolyma and Lena Rivers have varied over the observational periods available (Fig. 3). Spring river break-up occurs earlier in the season and clear patterns of increased winter discharge are apparent across both river catchments (Fig. 3a, b). Overall mean annual freshwater discharge has increased over the last decade (2010–2020) by 27.7% for the Kolyma River (94.6 to 120.7 km^3^ year^−1^) and 9.9% in the Lena River (626.9 to 689.1 km^3^ year^−1^) compared to a baseline period of 1971–2000 (black lines—Fig. 3).

Assuming climate projections of mean annual runoff increases of ~ 50% (± 25%) in the Kolyma River and 25% (+ 25%/− 20%) for the Lena River (Arnell, 2005; Shiklomanov et al. 2013; van Vliet et al. 2013; Koirala et al. 2014), we applied projections up to 2100 (Fig. 3c, d). A rapid increase in freshwater discharge since the 1971–2000 baseline meant future projections of + 25% on the Kolyma, or + 5% in the Lena, now represent a reduction in discharge relative to the freshwater loads observed over the last two decades (Fig. 3).

By 2100, we estimate annual mean discharge rates under these assumptions of 141.8 km^3^ year^−1^ (± 28.7 km^3^ year^−1^) and 783.6 km^3^ year^−1^ (± 81.9 km^3^ year^−1^) in the Kolyma and Lena Rivers, respectively.

### Role of permafrost OC composition on OC degradation rates

**Fig. 4:**
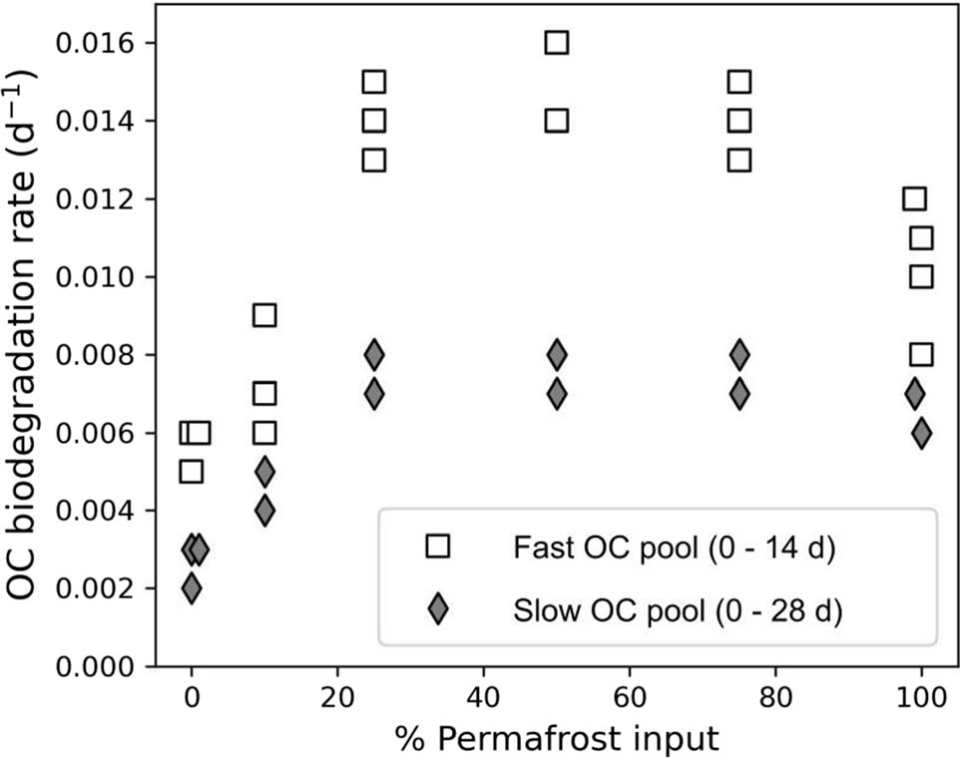
OC degradation rates in carbon-normalised Kolyma River waters with increasing percent permafrost-derived OC contributions. Fast and slow rates relate to OC losses measured over 14 and 28-day incubation periods, respectively. 0% permafrost input (= 100% Kolyma) represents contemporary mainstem waters, whereas 100% permafrost are permafrost and thaw stream derived waters. OC degradation rates have been normalised to September Kolyma mainstem in situ water temperature of 7.3 °C

Mean OC degradation rates in both the slow and fast OC pools increased relative to Kolyma mainstem rates (0% permafrost input: Fig. 4), with additions of permafrost-derived terrestrial OC (Fig. 4). Terrestrial OC degradation rates increased almost linearly with increasing permafrost OC contributions to the total DOC pool, up to approximately a 25% subsidy (Fig. 4). After approximately 25% of the total OC pool had been replaced by permafrost-derived OC, no further increases in bulk OC degradation rates were observed, and at very high permafrost-OC contributions (95%), degradation rates appeared to decline.

Our results demonstrate that increased OC degradation rates will be observed in waters receiving permafrost-thaw-derived OC, and that these increases were definitively due to compositional shifts in organic matter composition and not simply by concomitant increases in DOC concentrations. The levelling off and potential decline in OC degradation with permafrost-OC contributions greater than 25%, suggests additional constraints such as limited nutrient availability acted to limit faster terrestrial OC rates.

### OC degradation with permafrost subsidies and changing runoff

To combine our permafrost-OC experimental results above with previous studies, we collated and pooled data from published literature (Vonk et al. 2013; Mann et al. 2014, Table S3). To ensure data were comparable across studies, rates were binned into OC pools as above (rapid, fast, slow) and all normalised to 15 °C, an approximate nominal summer Kolyma mainstem surface water temperature.

**Fig. 5:**
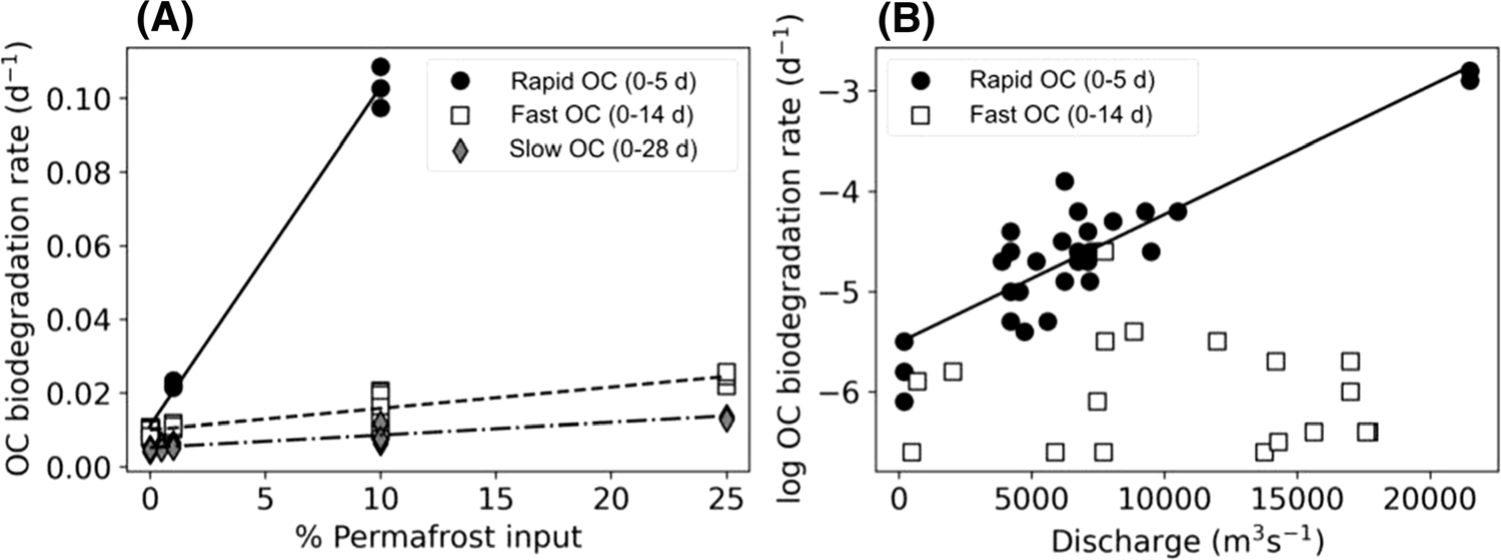
OC degradation rates in Kolyma River waters **a** calculated across all permafrost addition experiments with contributions up to and including 25% permafrost contributions (*n* = 55; normalised to 15 °C), and **b** determined in unamended waters and plotted on a log scale against river discharge. All rates have been corrected to in-situ temperature on sample date and discharge normalised to site location. All linear relationships shown are significant (*R*^2^ > 0.8, *p* < 0.0001). Full detail on linear regression fits provided in Table S4)

Mean OC degradation rates measured in all terrestrial pools were substantially faster with increasing permafrost-derived OC contributions (Table 2). Mean OC degradation rates increased by a factor of ten in the rapid OC pool (0.0093 to 0.1029 day^−1^) and doubled in the fast OC fraction (0.0046 to 0.0093 day^−1^), with a 10% subsidy to bulk OC pools. Small relative contributions of permafrost-derived OC (e.g., 1% of total OC) decreased overall OC lifetimes between 250% in the rapid OC pool to 125% in the fast OC fraction. Significant linear relationships (simple regression; *p* < 0.001) were found between increased permafrost-OC contributions up to 25%, and OC degradation rates in each OC fraction (Fig. 5a; *n* = 85; nominal 15 °C).

**Table 2:** OC degradation rates in experimental incubations of waters with up to 25% permafrost-thaw OC. Rapid OC fraction determined using oxygen loss measurements over 5-days. Fast and Slow OC pools are determined via dissolved OC loss over 14 or 28-days, respectively. All degradation rates were normalised to 15 °C, enabling comparison between experiments

Permafrost OC (%)	OC biodegradation rate (day^−1^)	OC lifetime (year^−1^)
Rapid OC pool		
0	0.0093 ± 0.0008	0.30 ± 0.02
1	0.0223 ± 0.0010	0.12 ± 0.01
10	0.1029 ± 0.0056	0.03 ± 0.001
Fast OC pool		
0	0.0091 ± 0.0010	0.31 ± 0.03
0.5	0.0103 ± 0.0003	0.27 ± 0.01
1	0.0112 ± 0.0007	0.25 ± 0.02
10	0.0163 ± 0.0047	0.18 ± 0.06
25	0.0239 ± 0.0020	0.11 ± 0.01
Slow OC pool		
0	0.0046 ± 0.0005	0.60 ± 0.06
0.5	0.0056 ± 0.0008	0.50 ± 0.08
1	0.0058 ± 0.0007	0.48 ± 0.06
10	0.0093 ± 0.0025	0.31 ± 0.09
25	0.0132 ± 0.0004	0.21 ± 0.01

To examine if changing hydrologic patterns influence bulk OC degradation rates within river catchments, we compare natural OC degradation rates reported above for the rapid (this study) and slow OC pools (Mann et al. 2012, 2015) with river discharge on that sample date. No relationship between OC rates in the slow turnover pool and discharge were found, but discharge was shown to be significantly and positively correlated with OC degradation rates of the rapid turnover pool (Fig. 5b; *R*^2^ = 0.82; Table S4).

This relationship most closely fit the equation:

3 $$\log k = 0.00013 \, \times {\text{discharge}} - 5.51246$$

where *log k* represents the log OC degradation rate in the rapid OC pool (day^−1^) and *discharg*e Kolyma River discharge (m^3^ s^−1^). The relationship was strongly influenced by extreme higher and lower OC rates measured in freshet waters (sampled during very high discharge) and under-ice waters (very low discharge conditions), respectively. This likely reflects the substantial shift in OC composition across the hydrograph (Mann et al. 2012).

## Discussion

### Terrestrial permafrost thaw and landscape evolution

The source and quantity of terrestrial OC mobilised from Arctic catchments will change in response to widespread landscape evolution due to climate warming. Both gradual and abrupt processes are taking place across river basins (Fuchs et al. 2020) releasing old permafrost-derived OC for decomposition and enabling its mobilisation and potential utilization within nearshore waters (Vonk and Gustafsson 2013). However, the rate of permafrost OC release to waters is dependent upon still uncertain projections of terrestrial permafrost thaw. The ice-rich permafrost across northeastern Siberia has been projected to remain relatively stable beyond 2100 even under extreme climate warming (RCP 8.5) (Koven et al. 2011, 2015), yet these estimates did not incorporate landforms such as thermokarst resulting from permafrost thaw, which are known to accelerate OC release substantially (Schneider von Deimling et al. 2015; Turetsky et al. 2020). A recent study has shown that substantial quantities of additional permafrost-derived OC thaw could occur in NE Siberia under future warming scenarios (Nitzbon et al. 2020). They show that when thermokarst-related permafrost thaw processes are included in models, a three-fold (RCP4.5) to 12-fold (RCP8.5) increase (compared to over previous projections) more OC can be thaw-affected to OC (Nitzbon et al. 2020).

Terrestrial OC collected from Pleistocene Yedoma permafrost have been found to be of good quality for future biological degradation (Haugk et al. in review). Both our studied rivers cut into extensive Yedoma deposits, like at the Sobo Sise cliff (Fuchs et al. 2020) and the Kurungnakh cliff (Stettner et al. 2018) on the Lena River, and the Duvanny Yar cliff (Strauss et al. 2012; Vonk et al. 2013) on the Kolyma River indicating that future landscape degradation or increased erosion and thermokarst in these catchments will liberate permafrost OC to nearshore environments.

### Permafrost thaw enhances aquatic OC degradation

Greater subsidies of permafrost-derived OC from land will increase mean degradation rates of OC in inland waters. We demonstrated that this was due to compositional shifts in the bulk OC pool, and irrespective of total DOC concentrations (Fig. 4). Our experimental results from waters collected during autumn months (e.g., 1% permafrost OC lifetime 0.38 year^−1^ at 7.3 °C) compare well with those previously reported in summer samples (1% permafrost OC lifetime 0.31 year^−1^ at 16.9 °C; Vonk et al. 2013), suggesting an enhanced degradation to OC from permafrost supply could be expected over the entire open water season.

Contrary to previous studies, OC degradation rates did not increase with additional permafrost-thaw contributions > 25% (Fig. 4) indicating that additional regulatory factors such as nutrient availability began to limit additional reactivity enhancements (Frey et al. 2009; Mann et al. 2014; Reyes and Lougheed 2015; Fouché et al. 2020). Associated enrichment of aquatic systems with nutrients from permafrost-derived OC additions could also therefore play an important role in determining future OC degradation rates. Linear increases in OC degradation rates with permafrost thaw contributions up to one-quarter of the total OC pool (Table 2), show that permafrost-derived OC additions will significantly enhance inland OC turnover over upcoming decades. Future thaw impacts may potentially be modelled using simple empirical relationships such as those we found (Fig. 5a), although additional research is needed across other Arctic catchments to confirm if similar relationships exist, especially across basins containing different permafrost types and formation histories.

Despite highly uncertain estimates for future terrestrial permafrost thaw, evidence is emerging to suggest the release of permafrost-derived OC to inland waters is underway (Mann et al. 2015; Abbott et al., 2015; Wickland et al., 2018; Wild et al. 2019; O’Donnell et al. 2020; Walvoord et al. 2020; Kokelj et al. 2020). Contemporary permafrost contributions to bulk Kolyma mainstem OC calculated using dual-isotopic (Δ^14^C/δ^13^C) signatures are estimated to be 0.7 ± 0.1% during August–September (Mann et al. 2015), and between 0.8 and 7.7% in late summer via a combination of ultrahigh-resolution mass spectrometry and ramped pyrolysis oxidation techniques (Rogers et al. 2021). The fraction of permafrost and peat deposits to total DOC within the Kolyma and Lena Rivers have also been estimated using Δ^14^C and source apportionment across seasons (Table S8 in Wild et al. 2019). Kolyma mainstem waters were estimated to contain between 4.6 to 18.7% (best estimate of 7.9%) of peat and permafrost during Spring, but up between 9.8 to 34.5% (16.3%) during winter months. Lena waters were estimated to contain 3.2 to 13.3% (best estimate of 5.6%) in spring and 6.9 to 25.4% (11.6%) during winter. The large differences in estimates between these studies demonstrate the difficulties in identifying permafrost contributions within river waters, although highlights relatively small current contributions, and suggest younger peat deposits contribute substantially to the bulk OC pool.

Using the relationship, we report between permafrost OC supply and increased OC degradation rates (Fig. 5a), we test the sensitivity of river OC to increased future permafrost supply. Assuming a conservative doubling of permafrost-derived OC to bulk river carbon pools (i.e., a further 0.7% permafrost contribution), we suggest mean OC degradation rates would increase from 0.0175 to 0.0240 day^−1^ in the rapid OC fraction and from 0.0055 to 0.0057 day^−1^ in the slow OC pool (Fig. 5a). These biolability rate increases translate to reductions in terrestrial OC lifetimes from 0.16 to 0.11 year^−1^ and 0.50 to 0.48 year^−1^, respectively. Increasing freshwater runoff will additionally transport terrestrial OC from upstream headwaters to mainstem river and coastal waters more rapidly (Catalán et al. 2016). Headwater catchments have an intimate link with the landscape and currently receive significantly greater proportions of permafrost-derived OC. For example, smaller streams within the Kolyma River were shown to contain 13 ± 4% of permafrost-derived OC and those affected by erosional processes 43 ± 21% (Table 1 in Mann et al. 2015). This material is currently rapidly processed within river networks reducing observed permafrost-derived OC contributions downstream (Mann et al. 2015; Spencer et al. 2015). More efficient delivery of permafrost-derived enriched OC from headwaters and tributaries may therefore significantly increase downstream degradation rates. As an example, if mainstem waters were to contain OC with 5.7% permafrost-derived OC as currently present within Kolyma minor tributaries (5.7 ± 3.5% permafrost contributions; Mann et al. 2015), degradation rates in the slow OC pool would increase from rates of 0.0055 day^−1^ (lifetime of 0.50 year^−1^; assuming current 0.7% permafrost subsidy), to 0.0072 day^−1^ (lifetime of 0.38 year^−1^). Associated increases in terrestrial OC degradation rates in upstream tributaries would also be expected, as they in turn receive greater subsidies from smaller headwater streams. It is however highly uncertain if mainstem waters will ever receive such subsidies, or how much they may make up of the bulk OC pool. Accurately constraining the amount of permafrost OC being released to headwaters, and improved methods for tracing permafrost OC through Arctic networks will be essential in understanding how permafrost underlain river catchments may adapt in response to future permafrost thaw and thermokarst events.

### Enhanced freshwater runoff increases aquatic OC degradation rates

Increasing freshwater runoff rates delivered greater quantities of terrestrial OC that could be rapidly degraded in aquatic ecosystems over the order of a few days (i.e., Rapid turnover OC; Fig. 5b). No comparable relationships between the rates measured in the ‘slow’ OC pools and discharge were identified (Fig. 5b). Increased freshwater discharge rates therefore appear to be associated with greater delivery of highly reactive OC from the landscape, likely fueling higher OC degradation rates in receiving stream and river waters. The lack of an empirical relationship between discharge and ‘fast’ or ‘slow’ OC pools suggest that the changing hydrologic runoff will not directly alter their degradation rates.

**Fig. 6:**
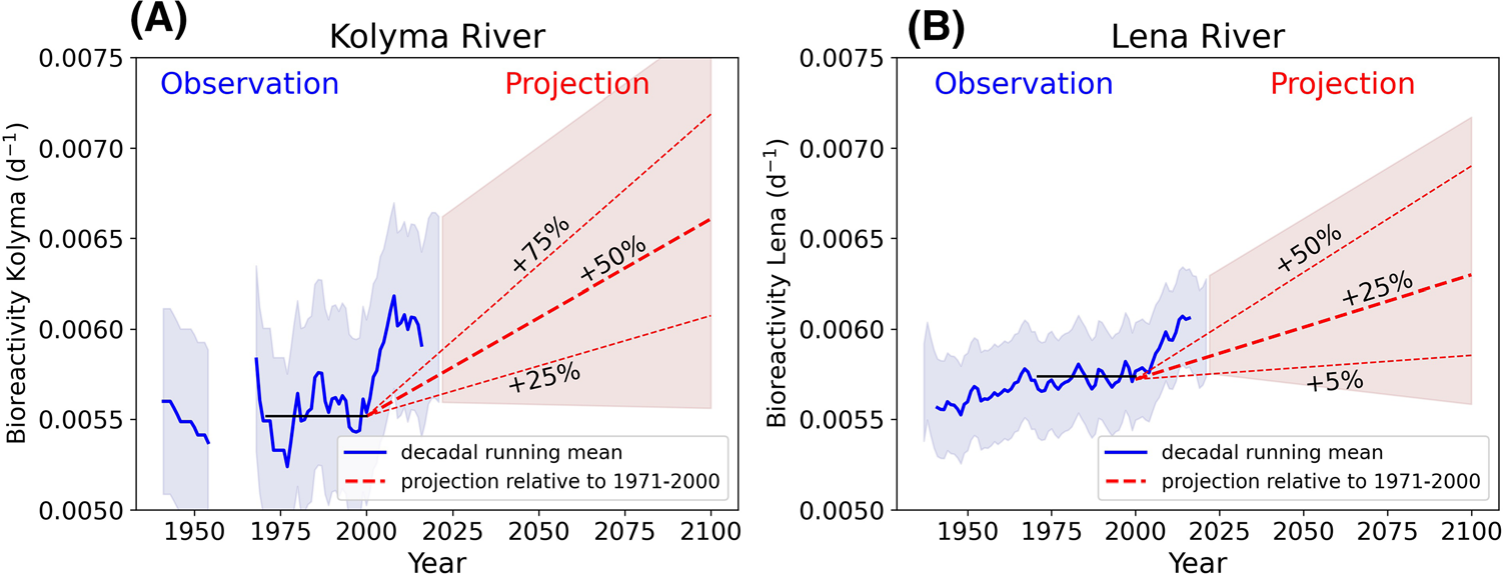
Observed and projected OC degradation rates (day^−1^) calculated using Eq. 2 for: **a** the Kolyma River and, **b** Lena River. OC degradation rates for the Lena River are scaled by calculating a scale factor (0.164) correcting for relative differences in discharge

Assuming the relationship between rapid OC pool degradation rates and discharge holds under future scenarios (Eq. 3), we apply this equation to discharge records from the Kolyma River (Fig. 3) to project how rapid OC pool degradation rates may change under future runoff patterns (Fig. 6). As noted above, OC pools in the Kolyma and Lena rivers are similar in composition (Mann et al. 2016) and thus we expect them to display comparable degradation rates as those reported in the Kolyma River. We therefore also examined how Lena River OC degradation rates may alter in response to increasing discharge but note that future studies are needed to test that these assumptions are valid. We scaled the Lena discharge to that of the Kolyma, using a scaling factor of 0.164 which was determined by dividing the mean annual Lena and Kolyma Rivers discharge. We then applied the scaled Lena River discharge to Eq. 3. Despite the many assumptions present in such calculations—especially in Lena River waters, it seems likely that an enhanced hydrological system will promote OC pools in river catchment that can be rapidly utilized by microorganisms.

Increased OC degradation rates in the ‘rapid’ turnover OC pool under future enhanced runoff conditions will likely fuel greater greenhouse gas emissions from Arctic catchments. For example, the Kolyma River mainstem is supersaturated in dissolved CH_4_ (15,300% relative to atmosphere) and CO_2_ (235%) fueling significant gas exchange fluxes from the river and gulf regions (Palmtag et al. 2021). Using a simple box model incorporating present-day runoff rates and field gas measurements, the authors estimate mean CH_4_ loads of 9.5 × 10^5^ kg CH_4_ year^−1^ enters the lower reach of the Kolyma River (ca. 100 km upstream of river mouth) during the open water period (1 Jun–1 Nov). Of these loads, they calculate losses of 49% (− 4.7 × 10^5^ kg CH_4_ year^−1^) to the atmosphere via gas exchange in the gulf, with total fluxes to the coastal ocean of 6.0 × 10^5^ kg CH_4_ year^−1^ (with net oxidation accounting for small variations). Assuming conservative increases in freshwater discharge of 25% and identical water gas concentrations, CH_4_ loads would be expected to increase to 11.9 × 10^5^ kg CH_4_ year^−1^, with gas exchange losses of 50% (− 6.0 × 10^5^ kg CH_4_ year^−1^) and fluxes to the ocean of 7.2 × 10^5^ kg CH_4_ year^−1^. These findings suggest that higher discharge rates have the potential to strengthen both greenhouse emissions from Arctic catchments as well as dissolved gas loads to coastal waters. Future work is therefore needed to understand how constituent river loads will increase under freshwater intensification.

Future decreasing ice thickness and broader sub-ice pathways will further increase the connectivity of Arctic rivers. This connectivity could account for increased winter runoff signals (Juhls et al. 2021) as observed here (Fig. 3a, b). Active layer thickening and Talik formations caused by warming may also cause increased connectivity and groundwater flow (Frey and McClelland 2009). This will lead to increasing subsurface water flow and greater leaching and contributions of old reactive permafrost-derived OC.

### How could future increases in the supply of OC from land impact coastal biogeochemistry?

Future changes in the quantity or composition of terrestrial OC delivered to the Arctic Ocean nearshore may play a significant role in shaping nearshore processes, largely through the supply of nutrients and terrestrial OC to coastal oceans. Increasing river discharge and coastal erosion across the Siberian Arctic is not only increasing terrestrial OC loads to coastal waters but is also likely to substantially alter its composition with greater subsidies of permafrost-derived OC translocated from river catchments (described above), and enhanced erosion of permafrost-rich coastlines (Günther et al. 2013). The future impact of terrestrial permafrost thaw and enhanced runoff rates on Arctic Ocean nearshore processes are however strongly influenced by estuarine removal processes, such as flocculation processes or biological or photochemical degradation before reaching the shelf. For example, only 5–15% of the particulate OC measured within the river mainstem is estimated to leave the Lena River delta (Semiletov et al. 2016). By contrast, a minimal removal of DOC (< 5%) was reported for a boreal river using a simple box model parameterized with river inputs, settling fluxes, advective export and solved for degradation (Gustafsson et al. 2000). This is in good agreement with the apparently linear and conservative mixing trends for DOC extending from the Lena River and into nearshore regions (Köhler et al. 2003; Amon 2004; Juhls et al. 2019), although these studies have historically only focused on late summer seasons. Further offshore, the inner and outer Lena-Laptev Sea plume has been shown to contain riverine DOC that is approximately two months old, having lost approximately 10% of the initial DOC (Alling et al. 2010). Substantial losses of DOC (ca. 10–20%) delivered by the Kolyma River into the East Siberian Sea have also been reported (Alling et al. 2010). Increasing exports of terrestrial OC therefore have the potential to be reflected in coastal nearshore environments and play a crucial role in affecting nearshore degradation rates.

Terrestrial lifetime estimates for the entire OC pool over the Laptev and East Siberian Shelf have previously been estimated from field dissolved OC measurements across the shelf, indicating lifetimes on the order of 3.3 year^−1^ (Alling et al. 2010) and 10 year^−1^ derived from ocean waters and used across the entire Arctic from a modelling study (Manniza et al. 2009). These are significantly longer than the lifetimes in contemporary Kolyma River mainstem waters calculated here which were on the order of 0.95 ± 1.3 year^−1^ (Slow OC pool; Table 1). Our results compare well with previous estimates of 0.7 year^−1^ in Alaskan rivers (Holmes et al. 2008). Increasing lifetime estimates reported from waters moving offshore are consistent with expected decreases in OC degradation rates across the aquatic-ocean continuum (Catalán et al. 2016). These changes appear not to be driven by the capabilities of the coastal microbial community, as parallel OC degradation rates measured in Kolyma River and coastal waters containing their natural microbial communities showed highly similar OC loss rates (Vonk et al. 2013). Future studies need to consider implementing different degradation rates for terrestrial OC throughout the nearshore, with faster rates within and near river mouths, and higher removal rate constants in Arctic shelf waters relative to the Arctic interior (Alling et al. 2010). The role of particulates across the nearshore also needs to be further understood, as adsorption and flocculation processes have the potential to change biodegradation rates and the ultimate fate of DOC (Keskitalo et al. in review).

Future contributions of permafrost-derived OC to coastal waters will additionally exacerbate reductions in bulk OC lifetimes across shelf waters. Rapid losses of fluvial permafrost OC within river catchments may cause limited quantities of permafrost OC to be exported to the nearshore, but as river catchments continue to degrade, and catchment OC residence times continue to decline, it is possible the composition of exported OC will shift. Direct inputs of particulate and dissolved permafrost-OC from increased coastal erosion may also increase (Jones et al. 2020). Here, we show that relatively small subsidies of permafrost could significantly increase degradation rates, with an additional 1% contribution to mainstem waters increasing OC loss rates by 20 to 60%, depending on the OC pool studied (Table 2). Enhanced coastal OC degradation could result in CO_2_ accumulation in coastal waters slowing or potentially reversing annual Arctic Ocean sea-air uptake and acting as positive feedback upon Arctic climate change. The Arctic Ocean is currently considered a small net sink of atmospheric CO_2_, with uptake estimates ranging between 0.1 to 0.2 Pg C year^−1^ (McGuire et al. 2009; Arrigo et al. 2010; Jeansson et al. 2011; Manizza et al. 2013; Schuster et al. 2013). Model estimates of coastal nearshore environments however often use only a single OC degradation rate to represent degradation rates across the entire Arctic Ocean (e.g., Manniza et al. 2009). Recent modelling efforts using a biogeochemical model incorporating terrestrial OC dynamics identifies the degradation rate of terrestrial OC as a critical parameter in projecting the strength and direction of future CO_2_ emissions from shelf waters (Polimene et al. submitted). The authors examined a range of OC lifetimes spanning 0.3 to 10 year^−1^ under changing terrestrial OC supply scenarios (+ 0 to 100% discharge) to the Laptev Sea and found that either increased OC loads or changing composition (reductions in OC degradation rates) significantly affected net shelf CO_2_ budgets. Furthermore, changes to terrestrial OC loads or composition to coastal waters had profound impacts upon light penetration, and in turn rates of primary production, as well as phytoplankton community dynamics. Recent suggestions that the riverine and erosional supply of terrestrial dissolved nitrogen may strengthen the Arctic shelf as a net CO_2_ sink (McGuire et al. 2010; Terhaar et al. 2021) may be optimistic. Changes to net primary production rates and phytoplankton community dynamics in shelf waters may also modify essential food webs and their distributions across changing Arctic coasts. Coastal food webs may also need to respond to enhanced rates of ocean acidification. The Arctic Ocean is particularly sensitive to ocean acidification due to the greater quantities of CO_2_ that can dissolve in cold waters and the changing alkalinity load received from Arctic Rivers (Drake et al. 2018a, b). Ocean acidification across the ESAS has been attributed to degradation of terrestrial organic matter and addition of CO_2_ rich waters from river runoff, rather than atmospheric CO_2_ uptake (Semiletov et al. 2016). Greater delivery of terrestrial materials, or any enhancement in OC degradation rates caused by increasing freshwater discharge or permafrost supply will, therefore, likely also cause a worsening of ocean acidification across coastal waters.

## Conclusion

We propose that nearshore regions across the Arctic are hotspots for environmental change requiring concerted and co-ordinated sampling efforts across river, estuary, coastal and shelf regions. An intensification of the hydrological cycle across the nearshore is underway and expected to continue well into the twenty-first century, with a range of complex and non-mutually exclusive impacts and greater dissolved organic carbon loads to coastal waters. Greater freshwater discharge rates may cause a lateral shift in terrestrial OC concentration and composition, efficiently translocating more biodegradable OC to mainstem and coastal waters for biodegradation or storage. Permafrost and peat-derived OC will be mobilised more rapidly into river networks from headwaters or via enhanced river erosion supplying an additional source of highly available OC to aquatic organisms, subsidising higher atmospheric greenhouse gas emissions during river transit and greater loads of dissolved concentrations to coastal waters. Coastal erosion will further increase permafrost OC pools in shelf waters. The rapidity of changes across the Arctic nearshore will require studies that incorporate new and existing observations with improved modelling efforts that can capture changing hydrology and coastal freshwater dynamics, as well as a range of terrestrial OC degradation rates. There is an explicit need to capture seasonal variability more effectively across all seasons, especially in underrepresented areas such as the Russian Arctic. Effective use of in-situ monitoring platforms and remote sensing products could aid in delivering spatially consistent data on OC fluxes, but it remains a challenge to “observe” permafrost OC mobilisation to the nearshore. Monitoring changes in bulk DOC degradation may prove a useful, and fundamentally viable metric to help monitor any shifts in fluvial and coastal OC amount and composition. Future increased quantities of terrestrial OC within coastal waters will cause a suite of physical and biogeochemical changes including in the availability of light and nutrients, patterns of ocean acidification and ultimately coastal productivity and fisheries.

## Societal and policy implications

Approximately, 10% of the 4 million people who live in the Arctic are Indigenous. The Arctic has been their home for thousands of years and over the millennia they have developed the skills to survive in areas of harshest living conditions and to adapt to changes. However, the rapid and unprecedented climatic and environmental changes that we are seeing in the Arctic today are the biggest long-term challenge that the Indigenous Peoples are facing. These changes are affecting indigenous practices such as reindeer herding, hunting, fishing, and gathering, ultimately challenging food security (Plate et al. 2021). Hydrological changes and permafrost degradation in the river catchments are affecting reindeer herding indigenous peoples who are dependent on the migration routes and pasture lands of the herd to maintain food security. Additionally, permafrost thaw related changes in riverine carbon and nutrient supply could affect fish stocks both in rivers and nearshore marine waters. Changes to the amount and type of marine plants (phytoplankton) may cause changes to the distribution, availability and biomass of coastal fish and higher mammals. Increased coastal erosion and permafrost inputs also has the potential to increase the concentration of contaminants—such as inorganic and methyl mercury, in inland and potentially coastal waters (St Pierre et al. 2018; Zolkos et al. 2020). This may result in greater loads of contaminants within coastal foods and accumulating up the food chain to higher species, resulting in greater risk to local peoples’ who rely on nearshore marine resources.

The Russian Arctic Rivers are important transportation routes both to supply the cities and settlements in the hinterland and to ship raw materials to the coastal zone and further via the Northern Sea Route. Port facilities and other infrastructure along the rivers and in the coastal and nearshore zone are vulnerable to an intensification of the hydrological cycle and to amplified permafrost degradation. Loss of nearshore sea-ice can be exacerbated by increasing coastal runoff and terrestrial loads (for instance through altering heat absorption into coastal waters). Greater volumes of shipping across Arctic coastal waters increases the risks of accidents and spillages across the nearshore, with the potential for long-term damage to coastal ecosystems and loss (or contamination) of essential species.

We, therefore, believe that this study’s topic is highly relevant for Arctic policymakers, in particular for the Arctic Council which promotes the cooperation between Arctic States, indigenous peoples and other Arctic residents with regard to sustainable development and environmental protection. The three Arctic Council working groups Conservation of Arctic Flora and Fauna (CAFF), Protection of the Arctic Marine Environments (PAME) and Sustainable Development Working Group (SDWG) as well as the Arctic Indigenous Peoples organizations, represented on the Council as Permanent Participants, are potential users of this study.

## Supplementary Information

Below is the link to the electronic supplementary material.

Supplementary file1 (PDF 262 KB)
