# Supplementary material for: Degrading permafrost river catchments and their impact on Arctic Ocean nearshore processes
Source: Ambio. 2021 Nov 30;51(2):439–55. doi: 10.1007/s13280-021-01666-z (PMC8692538; doi:10.1007/s13280-021-01666-z)
Supplement: Supplementary file 1 — Supplementary file1 (PDF 262 KB) [file 13280_2021_1666_MOESM1_ESM.pdf]

Ambio

Electronic Supplemental Material
This supplemental material has not been peer reviewed.

Title: Degrading permafrost river catchments and their impact on Arctic Ocean nearshore processes

Authors: Paul J. Mann, Jens Strauss, Juri Palmtag, Kelsey Dowdy, Olga Ogneva, Matthias Fuchs, Michael Bedington, Ricardo Torres, Luca Polimene, Paul Overduin, Gesine Mollenhauer, Guido Grosse, Volker Rachold, William V. Sobczak, Robert G. M. Spencer, and Bennet Juhls.

Sample date	OC fraction	Latitude (dd.dd)	Longitude (dd.dd)	Site corrected Discharge ($m^3 s^{-1}$)	Water T (°C)	Measured Bioreactivity *k* (d^{-1})	Temp Corrected Bioreactivity *k* (d^{-1})
13-Jul-11	Rapid	68.71957	158.67471	10500	16.9	0.0190	0.0153
15-Jul-11	Rapid	68.74053	161.28983	9490	16.1	0.0130	0.0099
17-Jul-11	Rapid	68.74648	161.30086	9290	15.6	0.0208	0.0153
20-Jul-11	Rapid	68.74034	161.2809	8060	16.1	0.0185	0.0141
21-Jul-11	Rapid	68.71628	158.68324	6250	17	0.0249	0.0202
22-Jul-11	Rapid	69.21184	161.4352	7350	16.9	0.0126	0.0102
23-Jul-11	Rapid	68.74174	161.28226	6250	16.9	0.0097	0.0078
03-Jul-12	Rapid	68.74528	161.29361	4730	11.2	0.0080	0.0043
04-Jul-12	Rapid	68.7386	161.27528	6150	11.3	0.0193	0.0106
06-Jul-12	Rapid	68.73187	161.27042	7110	12.7	0.0200	0.0121
07-Jul-12	Rapid	69.54577	161.93533	7220	12.8	0.0165	0.0100
07-Jul-12	Rapid	69.20985	161.43878	7110	13.2	0.0144	0.0090
08-Jul-12	Rapid	68.73417	161.26471	7190	13.8	0.0117	0.0076
08-Jul-12	Rapid	68.72260	158.69265	6750	13.8	0.0142	0.0092
09-Jul-12	Rapid	68.71973	161.26555	7110	14.7	0.0136	0.0094
09-Jul-12	Rapid	68.71967	161.28693	7110	14.2	0.0131	0.0088
10-Jul-12	Rapid	68.63368	161.27461	6750	15.7	0.0199	0.0148
10-Jul-12	Rapid	68.63673	161.26042	6750	14.3	0.0150	0.0101
10-Jul-12	Rapid	68.63927	161.26329	6750	14.2	0.0133	0.0089
12-Jul-12	Rapid	68.72163	158.68800	4530	15.2	0.0093	0.0067
14-Jul-12	Rapid	68.73594	161.27567	5600	15.4	0.0066	0.0048
17-Jul-12	Rapid	68.51748	160.96527	4201	16.2	0.0125	0.0096
18-Jul-12	Rapid	68.73901	161.28644	4201	15.5	0.0173	0.0127
19-Jul-12	Rapid	69.53667	161.91884	5170	14.2	0.0132	0.0088
21-Jul-12	Rapid	68.72879	161.27194	3872	15.3	0.0128	0.0092
15-May-11	Rapid	68.73594	161.27567	197	0.1	0.0089	0.0022
15-May-11	Rapid	68.73594	161.27567	197	0.1	0.0118	0.0030
15-May-11	Rapid	68.73594	161.27567	197	0.1	0.0155	0.0039
2011 freshet	Rapid	68.73594	161.27567	21500	5.5	0.1541	0.0564
2011 freshet	Rapid	68.73594	161.27567	21500	5.5	0.1726	0.0632
2011 freshet	Rapid	68.73594	161.27567	21500	5.5	0.1686	0.0617
18-Jul-12	Rapid	68.73901	161.28644	4201	15.5	0.0095	0.0070
18-Jul-12	Rapid	68.73901	161.28644	4201	15.5	0.0070	0.0051
18-Jul-12	Rapid	68.73901	161.28644	4201	15.5	0.0144	0.0105
26-Aug-12	Slow	68.73594	161.27567	7750	11.3	0.0090	0.0098[1]
31-Aug-12	Slow	68.73594	161.27567	8854	9.8	0.0048	0.0047[1]
14-Sep-12	Slow	68.73594	161.27567	7480	6.5	0.0028	0.0022[1]
09-Sep-13	Slow	68.73594	161.27567	14200	7.3	0.0039	0.0032[1]

12-Sep-13	Slow	68.73594	161.27567	12000	5.7	0.0056	0.0042[1]
19-Sep-13	Slow	68.73594	161.27567	7770	5.6	0.0058	0.0043[1]
19-May-10	Slow	68.73594	161.27567	494	0.3	0.0036	0.0013[2]
24-May-10	Slow	68.73594	161.27567	709	1.3	0.0073	0.0028[2]
25-May-10	Slow	68.73594	161.27567	2033	0.7	0.0081	0.0030[2]
27-May-10	Slow	68.73594	161.27567	5890	3.2	0.0031	0.0014[2]
28-May-10	Slow	68.73594	161.27567	7705	0.8	0.0037	0.0014[2]
29-May-10	Slow	68.73594	161.27567	13780	3.6	0.0031	0.0014[2]
30-May-10	Slow	68.73594	161.27567	15639	7.3	0.0027	0.0016[2]
31-May-10	Slow	68.73594	161.27567	17700	8	0.0027	0.0017[2]
01-Jun-10	Slow	68.73594	161.27567	17600	9.2	0.0026	0.0017[2]
03-Jun-10	Slow	68.73594	161.27567	17000	11.4	0.0033	0.0026[2]
04-Jun-10	Slow	68.73594	161.27567	17000	11.7	0.0043	0.0034[2]
07-Jun-10	Slow	68.73594	161.27567	14300	11	0.0019	0.0014[2]

Table S1. Kolyma River Site Location, discharge and OC degradation rates collected as part of POLARIS project (http://www.thepolarisproject.org/). [1]Data from Mann *et al.* 2015, [2]Data from Mann *et al.* 2012.

Percent permafrost addition	Sample collection date	OC fraction	Water T (ºC)	Temp Corrected Bioreactivity *k* (d^{-1})
0%	14-Jul-11	Rapid	16.1	0.0109[1]
0%	14-Jul-11	Rapid	16.1	0.0099[1]
0%	14-Jul-11	Rapid	16.1	0.0092[1]
1%	14-Jul-11	Rapid	16.1	0.0253[1]
1%	14-Jul-11	Rapid	16.1	0.0230[1]
1%	14-Jul-11	Rapid	16.1	0.0238[1]
10%	14-Jul-11	Rapid	16.1	0.1107[1]
10%	14-Jul-11	Rapid	16.1	0.1171[1]
10%	14-Jul-11	Rapid	16.1	0.1052[1]
0%	Jul-10	Fast	16.9	0.0121[2]
0%	Jul-10	Fast	16.9	0.0102[2]
0%	Jul-10	Fast	16.9	0.0100[2]
0.50%	Jul-10	Fast	16.9	0.0115[2]
0.50%	Jul-10	Fast	16.9	0.0120[2]
0.50%	Jul-10	Fast	16.9	0.0116[2]
1%	Jul-10	Fast	16.9	0.0134[2]
1%	Jul-10	Fast	16.9	0.0127[2]
1%	Jul-10	Fast	16.9	0.0135[2]
10%	Jul-10	Fast	16.9	0.0235[2]
10%	Jul-10	Fast	16.9	0.0232[2]
10%	Jul-10	Fast	16.9	0.0223[2]
100%	Jul-10	Fast	16.9	0.0242[2]
100%	Jul-10	Fast	16.9	0.0234[2]
100%	Jul-10	Fast	16.9	0.0233[2]
0%	Jul-10	Slow	16.9	0.0048[2]
0%	Jul-10	Slow	16.9	0.0058[2]
0%	Jul-10	Slow	16.9	0.0060[2]
0.50%	Jul-10	Slow	16.9	0.0070[2]
0.50%	Jul-10	Slow	16.9	0.0067[2]
0.50%	Jul-10	Slow	16.9	0.0053[2]
1%	Jul-10	Slow	16.9	0.0067[2]
1%	Jul-10	Slow	16.9	0.0075[2]
1%	Jul-10	Slow	16.9	0.0071[2]
10%	Jul-10	Slow	16.9	0.0134[2]
10%	Jul-10	Slow	16.9	0.0130[2]
10%	Jul-10	Slow	16.9	0.0134[2]
100%	Jul-10	Slow	16.9	0.0164[2]
100%	Jul-10	Slow	16.9	0.0144[2]
100%	Jul-10	Slow	16.9	0.0146[2]

Table S2. Temperature corrected OC degradation rates with permafrost-derived OC contributions. [1]Data from Mann *et al.* 2014, [2]Data from Vonk *et al.* 2013.

Percent permafrost addition	Sample collection date	OC fraction	Temp normalised Bioreactivity *k* (d^{-1})*
0	14-Jul-11	Rapid	0.0101[1]
0	14-Jul-11	Rapid	0.0092[1]
0	14-Jul-11	Rapid	0.0086[1]
1	14-Jul-11	Rapid	0.0234[1]
1	14-Jul-11	Rapid	0.0214[1]
1	14-Jul-11	Rapid	0.0221[1]
10	14-Jul-11	Rapid	0.1026[1]
10	14-Jul-11	Rapid	0.1085[1]
10	14-Jul-11	Rapid	0.0974[1]
0	Jul-10	Fast	0.0106[2]
0	Jul-10	Fast	0.0089[2]
0	Jul-10	Fast	0.0088[2]
0.5	Jul-10	Fast	0.0100[2]
0.5	Jul-10	Fast	0.0105[2]
0.5	Jul-10	Fast	0.0102[2]
1	Jul-10	Fast	0.0117[2]
1	Jul-10	Fast	0.0112[2]
1	Jul-10	Fast	0.0118[2]
10	Jul-10	Fast	0.0206[2]
10	Jul-10	Fast	0.0204[2]
10	Jul-10	Fast	0.0195[2]
100	Jul-10	Fast	0.0212[2]
100	Jul-10	Fast	0.0205[2]
100	Jul-10	Fast	0.0204[2]
0	Jul-10	Slow	0.0042[2]
0	Jul-10	Slow	0.0051[2]
0	Jul-10	Slow	0.0053[2]
0.5	Jul-10	Slow	0.0062[2]
0.5	Jul-10	Slow	0.0059[2]
0.5	Jul-10	Slow	0.0047[2]
1	Jul-10	Slow	0.0059[2]
1	Jul-10	Slow	0.0066[2]
1	Jul-10	Slow	0.0062[2]
10	Jul-10	Slow	0.0117[2]
10	Jul-10	Slow	0.0114[2]
10	Jul-10	Slow	0.0117[2]
100	Jul-10	Slow	0.0144[2]
100	Jul-10	Slow	0.0126[2]
100	Jul-10	Slow	0.0128[2]

0	09-Sep-13	Fast	0.0100
0	09-Sep-13	Fast	0.0078
0	09-Sep-13	Fast	0.0083
1	09-Sep-13	Fast	0.0102
1	09-Sep-13	Fast	0.0109
10	09-Sep-13	Fast	0.0095
10	09-Sep-13	Fast	0.0161
10	09-Sep-13	Fast	0.0119
25	09-Sep-13	Fast	0.0218
25	09-Sep-13	Fast	0.0245
25	09-Sep-13	Fast	0.0256
50	09-Sep-13	Fast	0.0266
50	09-Sep-13	Fast	0.0233
50	09-Sep-13	Fast	0.0269
75	09-Sep-13	Fast	0.0253
75	09-Sep-13	Fast	0.0230
75	09-Sep-13	Fast	0.0240
99	09-Sep-13	Fast	0.0203
99	09-Sep-13	Fast	0.0206
99	09-Sep-13	Fast	0.0211
100	09-Sep-13	Fast	0.0183
100	09-Sep-13	Fast	0.0176
100	09-Sep-13	Fast	0.0140
0	09-Sep-13	Slow	0.0044
0	09-Sep-13	Slow	0.0040
0	09-Sep-13	Slow	0.0046
1	09-Sep-13	Slow	0.0051
1	09-Sep-13	Slow	0.0050
10	09-Sep-13	Slow	0.0064
10	09-Sep-13	Slow	0.0071
10	09-Sep-13	Slow	0.0077
25	09-Sep-13	Slow	0.0132
25	09-Sep-13	Slow	0.0136
25	09-Sep-13	Slow	0.0127
50	09-Sep-13	Slow	0.0142
50	09-Sep-13	Slow	0.0144
50	09-Sep-13	Slow	0.0127
75	09-Sep-13	Slow	0.0124
75	09-Sep-13	Slow	0.0127
75	09-Sep-13	Slow	0.0129
99	09-Sep-13	Slow	0.0115
99	09-Sep-13	Slow	0.0117
99	09-Sep-13	Slow	0.0122

100	09-Sep-13	Slow	0.0100
100	09-Sep-13	Slow	0.0094

Table S3. Temperature corrected OC degradation rates with permafrost-derived OC contributions.
*Temperature corrections were calculated at a fixed temperature of 15°C to allow comparisons between studies.
[1]Data from Mann *et al.* 2014, [2]Data from Vonk *et al.* 2013.

	Terr-OC pool	*n*	R^2	slope	intercept	p value	std_err
a) Permafrost additions	Rapid	*9*	*0.99*	*0.00919304029*	*0.0111032967033*	*3.86e-09*	*0.0002610*
	Fast	*23*	*0.80*	*0.00057777292*	*0.0099881671903*	*8.35e-09*	*6.29e-05*
	Slow	*23*	*0.81*	*0.00033981412*	*0.0052387199670*	*4.99e-09*	*3.58e-05*
b) Discharge	Rapid	*34*	*0.82*	*0.00012798218*	*-5.512462638969*	*1.88e-13*	*1.06e-05*

Table S4. Significant linear relationships between a) permafrost % contributions and bioreactivity (*k*) in each OC pool and b) discharge ($m^3\ s^{-1}$) and log bioreactivity (log *k*) in the rapid turnover OC pool.
